# Early-stage immunoexpression of inflammatory, angiogenic, and survival markers in tongue epithelium of mice exposed to hookah smoke

**DOI:** 10.1371/journal.pone.0314794

**Published:** 2025-02-26

**Authors:** Aldini Beuting Pereira, Arieli Carini Michels, Sarah Freygang Mendes Pilati, Filipe Modolo, Ana Paula Camargo Martins, Caroline Busatta Vaz De Paula, Seigo Nagashima, Lúcia Noronha, Ana Clara Prado Fonseca, Ariane Jéssica Torres Turmina, Heloisa Franco De Meira, Luisa Gabriela Carneiro Ramos, Thaís Grupp Da Rosa, Thais Maria Dos Santos Eckhardt, Sérgio Aparecido Ignácio, Luciana Reis Azevedo Alanis, Paulo Henrique Couto Souza, Rodrigo Nunes Rached, Bruno Correia Jham, Everdan Carneiro, Emanuela Carla Dos Santos, Aline Cristina Batista Rodrigues Johann

**Affiliations:** 1 Programa de Pós Graduação em Odontologia, Escola de medicina e Ciências da Vida, Pontifícia Universidade Católica do Paraná, Curitiba, Paraná, Brazil; 2 Faculdade de Odontologia Universidade do Vale do Itajaí, UNIVALI, Itajaí, Santa Catarina, Brazil; 3 Departamento de Patologia Universidade Federal de Santa Catarina, Florianópolis, Santa Catarina, Brazil; 4 College of Dental Medicine-Illinois, Midwestern University, Downers Grove, Illinois, United States of America; Università degli Studi della Campania, ITALY

## Abstract

**Objective:**

The objective was to evaluate the early-stage immunoexpression of markers (COX-2, NF-kB, VEGFR-1 and apoptotic index) related to inflammation, angiogenesis, and cell survival in the tongue dorsum epithelium of mice exposed to hookah smoke.

**Materials and methods:**

The sample consisted of Swiss mice (N = 20), female gender, aged 2 months, and approximately 25g each, four groups (n = 5) mice: group exposed to fresh air and groups exposed to hookah smoke for 7, 15, and 30 days. Tongues were embedded in paraffin. A tissue microarray was constructed, and immunohistochemistry was performed for Cyclooxygenase 2, NF-kappa B, Vascular Endothelial Growth Factor Receptor 1, and terminal deoxynucleotidyl transferase dUTP nick-end labeling (TUNEL) for apoptotic analysis. The positive and negative cells were quantified in the epithelium of the mid-dorsal tongue region. Kruskal-Wallis and Dunn tests was made.

**Results:**

The apoptotic index was higher at 30 days of smoke exposure (20.38% basal/ 19.63%/ suprabasal) compared to the group exposed to air (9.55%/ 11.88%), The expression of Vascular Endothelial Growth Factor Receptor 1 was higher at 30 days of smoke exposure (30.15%/ 38.15%) compared to the group exposed to air (18.25%/ 3.60%).

**Conclusion:**

Hookah smoke induced greater apoptosis and increased expression of Vascular Endothelial Growth Factor Receptor 1 in the epithelium of the tongue at 30 days, potentially playing a role in the initial stages of carcinogenesis, in the early stages of hookah use.

## Introduction

The hookah is a device used for tobacco smoking, in which the charcoal-heated air passes through the tobacco, and its resultant smoke goes through a water filter prior to inhalation [1]. The utilization of hookah has been linked to tobacco-related diseases, emerging as a public health concern according to the World Health Organization (WHO) [2].

The elevated temperature of the hookah can lead to the generation of diverse noxious chemical compounds [3], and its smoke can contain substantial concentrations of nicotine and heavy metals [4]. This exposure to hookah can engender health hazards [5], owing to the presence of numerous toxics and potentially carcinogenic compounds within hookah smoke. As such, there is a significant propensity for hookah usage to serve as a potential risk factor for the development of potentially malignant and malignant oral lesions [6]. In addition to these conditions, hookah usage has also been linked to periodontitis, dry socket [7], and peri-implant diseases [8].

One way to assess the potential deleterious effects of hookah usage is through immunohistochemical analysis, as the expression of tissue proteins may undergo alteration due to exposure to compounds present in the smoke. There exist those proteins that have been previously scrutinized in the context of potentially malignant and malignant oral lesions: cyclooxygenase 2 (COX-2), NF-kappa B (NF-kB) [9], those related to apoptosis [10], and Vascular Endothelial Growth Factor Receptor 1 (VEGFR-1) [11].

COX-2 is an enzyme activated in response to inflammatory stimuli and can mediate pro-tumorigenic effects through cellular proliferation, inhibition of apoptosis, and promotion of angiogenesis by inducing vascular endothelial growth factors [12]. NF-kB plays a pivotal role in tumorigenesis, as it governs the expression and function of numerous genes involved in processes such as autonomous growth, insensitivity to anti-proliferative signals, evasion of apoptosis, limitless replicative potential, neoangiogenesis, tissue invasion, and metastasis [13].

Apoptosis, the process of programmed cell death [14], corresponds to a physiological mechanism that eliminates cells, playing a crucial role in maintaining cellular homeostasis, proliferation, and differentiation. Disruptions in the process of cell death can lead to uncontrolled tumor growth and facilitate tumor formation [15]. One method to identify apoptosis is through the terminal deoxynucleotidyl transferase dUTP nick-end labeling (TUNEL) technique [14]. Vascular Endothelial Growth Factor Receptor 1 (VEGFR-1 or Flt-1), a tyrosine kinase receptor, holds significant relevance in tumor-associated angiogenesis [16] and mediates mitogenic functions [17].

By studying these markers together, it is possible to gain an integrated view of the molecular pathways governing inflammation, angiogenesis, and cell survival. This holistic approach can identify potential therapeutic targets and more effective treatment strategies, contributing to advances in cancer research and clinical management.

Considering the potential implications of COX-2, NF-kB, VEGFR-1, and apoptosis in carcinogenesis, coupled with the carcinogenic propensity of compounds discharged during hookah consumption, and the lack of investigations scrutinizing these biomarkers and the apoptotic index in the tongues of mice subjected to hookah smoke, this study is justified.

The objective of this study was to evaluate the early-stage immunoexpression of markers (COX-2, NF-kB, VEGFR-1 and apoptotic index) related to inflammation, angiogenesis, and cell survival in the tongue dorsum epithelium of mice exposed to hookah smoke. The null hypothesis posits that there are no differences in the expression of COX-2, NF-kB, VEGFR-1, and the apoptotic index in the epithelium of the mid-dorsal tongue region of mice following exposure to hookah smoke, as compared to exposure solely to fresh air.

## Materials and methods

The study was observational and cross-sectional, approved by the Animal Ethics Committee Universidade do Vale do Itajaí (Univali-CEUA): 063/17.

The experimental phase involving mice was conducted at the Teaching Institution of the Universidade do Vale do Itajaí. In relation to the mice, appropriate measures were taken to minimize pain or discomfort [18], while the immunohistochemistry procedures were performed.

The sample consisted of Swiss mice (N = 20), female gender, aged 2 months, and approximately 25g each, all animals were assessed by the veterinarian as healthy. The mices were housed in conventional cages and maintained on a 12-hour light-dark cycle with a daily period for food and water exchange. The replacement of water, food, and bedding was standardized for all groups and the room was kept at a stable temperature of 22°C. The animals were removed from the Central Animal Facility one week before the experiment for acclimatization in the Experimental Laboratory Animal Facility.

All the mices in each group were of the same species, sex, age, and weight. Therefore, the mices were randomly distributed among the groups, ensuring that there was no differentiation due to intervening variables.

Besides these criteria, no other criteria were used for including or excluding animals during the experiment. The mice were randomly divided into four groups of five animals each, the sample size was decided based on convenience, in accordance with the availability of animals and reagents. There were no exclusions and there are no reports of any expected or unexpected adverse event. No animal was lost or suffered and any endpoint.

For each analysis, the exact value of n in each experimental and control group were five mice each, due to the size of the box and the flow of the pump used to expose the hookah smoke. There was no randomization method, nor were the confounders controlled. All cages were labelled and identified by group to prevent any exchange between the groups. Exposure was always randomized and blinded to the researchers between the experimental groups.

One research aware of the group allocation at the different stages of the experiment (during the allocation, the conduct of the experiment, the outcome assessment, and the data analysis).

One of the groups was the control group exclusively exposed to fresh air, while the remaining three groups underwent whole-body exposure to hookah smoke for 7, 15, and 30 days, with each session lasting 30 minutes daily. This control group was handled under identical conditions except for the exposure to smoke to rule out other environmental factors affecting the results. This exposure regimen involved a sequence of 2 seconds of direct exposure to hookah smoke, followed by a subsequent 58-second period of exposure to fresh air. This protocol was executed in alignment with the established methodology [19]. This exposure occurred through the placement of mice within a hermetically sealed glass chamber measuring 175x170x270mm, secured with silicone, featuring a 4mm where a silicone tube was positioned. The humane endpoints and the signs were monitored visually and daily. The smoke components would accumulate on the mice’s fur, and tongue exposure occurred because of their self-grooming behavior [18].

The mice’s exposure duration to the smoke was selected based on published investigations that scrutinized the cardiorespiratory repercussions of hookah smoke in human subjects [20,21]. In order to calibrate the electric suction apparatus, a calculation proportionate to the weight and size equivalent to five mice present within the glass chamber was executed, the electric suction machine was adjusted to result in a total volume of 530 ml, as the Beirut method prescribes [22].

The utilized hookah belonged to the Mizo brand (Al Nakhla Tobacco Company - Free Zone S.A. E®, Shibin El Kom, Egypt), containing an unprocessed tobacco percentage of 0.5%. The charcoal utilized was Bamboo Brasil Hookah Charcoal (Egitape Importação e Exportação LTDA®, São José, Santa Catarina), with dimensions of 2 cm × 2 cm.

Following the last exposure session for each group on their respective days, the animals were euthanized through anesthetic overdose using 50 µ L of Xylazine (0.23g/ml) (Ceva, Paulínia, Brazil) and 210 µ L of Ketamine (0.1g/ml) (Ceva, Paulínia, Brazil) per 10 grams of the animal’s weight. Sample collection was achieved through surgical removal of the tongue, which was transversely sectioned across the epithelium at the mid-dorsal region of the tongue. There was blinding during the allocation, conduct of the experiment, and outcome assessment among the researchers who carried out the research with the animals, but the researcher in charge knew the identifications. The control group was also labelled during exposure to ensure that there was no exposure to smoke. The studied tissues were fixed in 4% paraformaldehyde (Synth, Diadema, Brazil) in pH 4 to 7 phosphate buffer (Dinâmica, Indaiatuba, Brazil). The tissue was dehydrated in solutions of increasing concentrations of ethyl alcohol. Subsequently, it was cleared with xylene, followed by impregnation with histological paraffin, (Alphatec, São José do Pinhais, Brazil), and embedded in a paraffin block.

A tissue microarray (TMA) procedure was conducted. A 3mm diameter cylinder was extracted from a donor block using a trephine drill (Neodent, Curitiba, Brazil) coupled with a 130-watt suspension motor provided by Bethil (Prometal Ind. LTDA, Marília, Brazil). These cylindrical samples were methodically organized into rows and columns and subsequently embedded within paraffin (receiving block) [23,24].

### Immunohistochemical analysis

#### COX-2, NF-kB, and VEGFR-1.

Histological sections of four-micrometer thickness were obtained from the paraffin blocks that underwent deparaffinization using xylene, followed by sequential hydration through a series of decreasing alcohol concentrations, and then incubated in a 5% solution of hydrogen peroxide and methanol (Biotec, Curitiba, Brazil) to block endogenous peroxidase activity. For antigen retrieval, the slides were subjected to immersion in Immuno Retriever (Dako, Carpinteria, CA), followed by incubation with primary antibodies at 4°C within a humid chamber overnight:

- COX-2 (E3034, Spring Bioscience, California, USA) at a dilution of 1:200.
- NF-kB (ab7971, Abcam, Cambridge, UK) at a dilution of 1:200.
- VEGFR-1 (PA-211731, Thermo Fisher Scientific, Massachusetts, USA) at a dilution of 1:50.

Detection was carried out using Advance Link, followed by Advance Enzyme, both for 30 minutes at room temperature within a humid chamber (Dako Corporation, Carpinteria, CA, USA, code K406889). The reaction was visualized using a chromogenic solution of 3,3’-diaminobenzidine (DAB - Sigma Chemical, St. Louis, USA, code D7679), and counterstaining was performed with Harris hematoxylin solution (Biotec, Curitiba, Brazil). Subsequent processing included dehydration in ethanol and clarification in xylene. To affirm reaction efficacy, positive controls encompassing VEGFR-1 (injured artery), NF-kB (breast neoplasia), and COX-2 (colon neoplasia) were employed. A negative control was also implemented, involving omission of the primary antibody in one of the sections.

#### TUNEL.

The TUNEL technique was conducted to detect apoptosis in epithelial cells. This method is based on labeling DNA fragments resulting from genomic DNA fragmentation. The *In-situ* Cell Death Detection Kit, POD - Roche® (Roche Diagnostics GmbH, 11684817910, Mannheim, Germany) was employed for this purpose, following the manufacturer’s instructions meticulously.

Following the deparaffinization of the sections, endogenous peroxidase activity was inhibited using a 5% hydrogen peroxide and methanol solution. The sections were then subjected to antigen retrieval using citrate buffer at pH 6.0 to facilitate the recovery of antigenic epitopes.

The slides were coated with a mixture containing enzyme solution and label solution, and then incubated in a humid and dark environment. Subsequently, the slides were treated with converter-POD and incubated within a humid chamber. The slides were incubated in a diaminobenzidine (DAB) solution (K3468 DAKO DAB +  Chromogen Substrate Liquid System, Carpinteria, CA, USA). They were counterstained with Harris hematoxylin. In negative controls, the enzyme solution was omitted. The positive control consisted of oral squamous cell carcinoma (OSCC).

### Immunostaining analysis

There was blinding during immunohistochemistry analysis and quantification. The slides were digitized, and the images were analyzed at 200x magnification using the ZEN 2.3 lite software (ZEISS Microscope Software ZEN Lite). The “events” tool was employed to obtain the count of positive and negative epithelial cells in the basal and suprabasal layers, considering the nucleus and/or cytoplasm, within the mid-dorsal portion of the tongue. A thorough image scan was conducted until a total of one thousand cells (positive +  negative) were included in the analysis. Cells exhibiting brown-stained nucleus and/or cytoplasm were classified as positive. For COX-2 evaluation, cytoplasmic staining was considered [25], NF-kB in both nucleus and cytoplasm [26], apoptotic index in the nucleus [27], and VEGFR-1 within the cytoplasm [28].

To assess reproducibility, a recount tally of all images for the COX-2, NF-kB, VEGFR-1 markers, and the apoptotic index was conducted after a 21-day interval. Each marker and the apoptotic index were assessed by a sole observer. Considering the three markers and the apoptotic index, with twenty mice per marker, a thousand cells were enumerated for each mouse, culminating in a cumulative count of 160,000 cells. The outcome measures assessed were the percentage of cells positive for COX-2, NF-kB, and VGFR-1, and the apoptosis index.

### Statistical analysis

The data were analyzed using SPSS software version 25.0 (SPSS, Inc., Chicago, IL, USA). The adopted significance level was 5%. The intraclass correlation coefficient was excellent across all variables, ranging from 0.79 to 0.99. No systematic error was observed, as all p-values were >  0.05.

For the comparative analysis of the dependent variables COX-2, NF-kB, VEGFR-1, and the apoptotic index in the epithelium of the dorsal tongue, within the nucleus and/or cytoplasm, in the basal and suprabasal layers, among groups exposed to fresh air and those exposed to hookah smoke for seven, fifteen, and thirty days, the chosen test was the non-parametric Kruskal-Wallis test, followed by the non-parametric pairwise Dunn test for comparisons. In the present study, we applied the non-parametric Kruskal-Walli’s test because there were four groups to analyze, and the data did not follow a normal distribution according to the Shapiro-Wilk test. Additionally, the samples were independent and not paired. The Dunn test assumption is to compare independent samples when the data do not exhibit a normal distribution. The Kruskal-Walli’s test indicated that there was a difference between at least two or more groups, leading us to apply the Dunn test to identify which groups differed from each other. This test is appropriate when we have independent samples, and the data do not follow a normal distribution. To assess the sample power, an analysis of variance based on rank (ANOVA) test was conducted. In all tests, the significance level was set at p <  0.05.

## Results

The immunexpression of COX-2 and NF-kB showed no statistical differences among the groups (Table 1).

**Table 1:** Median percentage and interquartile range of positive cells in the nucleus and/or cytoplasm of the basal and suprabasal layers for COX-2, NF-kB, VEGFR-1, and the apoptotic index in the epithelium of the mid-dorsal tongue region of mice exposed solely to fresh air and those exposed to hookah smoke for seven, fifteen, and thirty days.

	Fresh air (median / AIQ)	7 days of exposure to hookah smoke (median / AIQ)	15 days of exposure to hookah smoke (median / AIQ)	30 days of exposure to hookah smoke (median / AIQ)	Kruskal-Wallis (p)	Statistical power
**COX-2**						
Basal	1.61% / 3.65	0.65% / 1.61	0.00% / 5.05	2.22% / 4.94	0.24	–
Suprabasal	1.72% / 3.89	0.65% / 1.99	0.00% / 7.12	2.12% / 3.16	0.24	–
**NK-kB nucleus**						
Basal	0.00% / 0.07	0.00% / 0.00	0.00% / 0.00	0.00% / 0.00	0.09	–
Suprabasal	0.05% / 0.15	0.00% / 0.02	0.00% / 0.00	0.00% / 0.05	0.16	–
**NF-kB cytoplasm**						
Basal	0.35% / 0.70	0.55% / 1.37	0.08% / 0.23	0.00% / 0.10	0.09	–
Suprabasal	0.10% / 0.55	0.25% / 2.02	0.18% / 0.64	0.08% / 1.48	0.55	–
**Apoptotic index**						
Basal	9.55% / 0.80 A	9.35% / 1.85 A	10.80% / 3.65 A	20.38% / 1.51 B	**0.00**	0.98
Suprabasal	11.88% / 1.60 A	16.25% / 7.78 AB	14.40% / 4.94 A	19.63% / 0.86 B	**0.01**	0.95
**VEGFR-1**						
Basal	18.25% / 9.95 A	16.05% / 3.95 AB	25.81% / 6.53 AC	30.15% / 8.63 C	**0.00**	0.99
Suprabasal	3.60% / 10.25 A	10.80% / 3.20 A	39.36% / 17.20 B	38.15% / 12.90 B	**0.00**	0.99

Nonparametric Dunn’s test. Different letters indicate statistically significant differences. Basal apoptotic index: fresh air × 30 days p = 0.00; 7 days × 30 days p = 0.00; 15 days × 30 days p = 0.04. Suprabasal apoptotic index: fresh air × 30 days p = 0.00; 15 days × 30 days p = 0.03. Basal VEGFR-1: fresh air × 30 days p = 0.00; 7 days × 15 days p = 0.03; 7 days × 30 days p = 0.00. Suprabasal VEGFR-1: fresh air × 15 days p = 0.00; fresh air × 30 days p = 0.00; 7 days × 15 days p = 0.00; 7 days × 30 days p = 0.01. One-way ANOVA based on rank p < 0.05.

**Fig 1:**
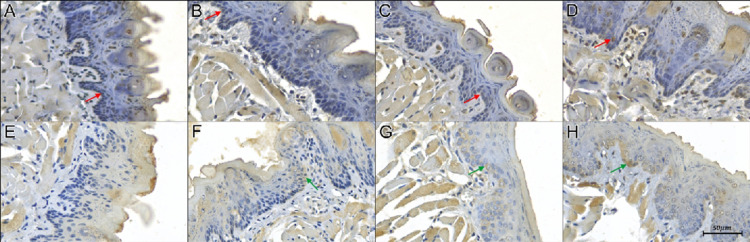
Photomicrograph in the middle portion of the dorsal tongue mucosa of mice subjected to hookah smoke in the following groups. 1) **revealing apoptotic cells** (A) fresh air exposure; (B) experimental group with seven days of exposure; (C) experimental group with fifteen days of exposure; (D) experimental group with thirty days of exposure to hookah smoke, at a magnification of 200x, TUNEL assay. Red arrows indicate nuclear immunostaining; 2) **expression of VEGFR-1** (E) fresh air exposure; (F) experimental group with seven days of exposure; (G) experimental group with fifteen days of exposure; (H) experimental group with thirty days of exposure to hookah smoke, at a magnification of 200x. Green arrows indicate cytoplasmic immunostaining.

The apoptotic index in the basal layer exhibited greater values after thirty days of exposure to hookah smoke in comparison to the other experimental groups. In the suprabasal layer, the apoptotic index displayed higher values after thirty days of exposure, when compared to the group exposed only to clean air and to fifteen days of exposure to hookah smoke (Table 1 and Fig 1). The statistical power for the basal layer reached 0.98, while in the suprabasal layer, it achieved a level of 0.95.

The VEGFR-1 expression in the basal layer exhibited elevated levels after thirty days of exposure, in contrast to the group exposed to fresh air and those subjected to seven days of hookah smoke exposure. Similarly, in the suprabasal layer, the expression of VEGFR-1 demonstrated increased levels after thirty days of exposure, compared to the group exposed only to clean air and to seven days of hookah smoke exposure (Table 1 and Fig 1). The statistical power for both the basal and suprabasal layers reached 0.99.

## Discussion

This study assessed COX-2, NF-kB, VEGFR-1, and the apoptotic index in the midsection of the dorsal tongue of mice exposed to hookah smoke. The null hypothesis was rejected as hookah smoke induced greater apoptosis in the tongue after 30 days, which was also observed for VEGFR-1. In the latter case, a substantial upsurge becoming evident starting from the 15th day of exposure.

The present study did not show differences between the groups regarding COX-2 and NF-kB. However, previous studies have observed a gradual increase in COX-2 expression in normal oral tissues, potentially malignant lesions, and OSCC associated with tobacco use [9]. Furthermore, there was an increase in expression from normal oral tissues to oral leukoplakia (OL) and oral cancer [25]; superexpression was noted in OL compared to normal oral mucosa [29], as well as high expression in OL and head and neck squamous cell carcinoma [30,31].

A gradual increase in NF-kB expression was observed in normal oral tissues, potentially malignant lesions, and OSCC associated with tobacco use [9]. This increase was also noted from normal oral mucosa to hyperplasias, dysplasias, and OSCC [26]. Additionally, there was a progressive increase in NF-kB expression from normal oral tissue to OL and oral cancer [25].

In the studies mentioned earlier, the predominant mode of tobacco exposure mostly pertains to conventional cigarette smoking, which is subject to regulatory control over the concentrations of its constituents. In contrast, for hookah smoking, such regulatory oversight is absent, leading to variable concentrations of its compounds that deviate from those present in conventional cigarettes [32]. This variability could potentially contribute to the differing outcomes observed in studies like ours, particularly concerning COX-2 and NF-kB expressions. Another plausible explanation is that the present study assessed exposure durations of 7, 15, and 30 days, whereas the exposure durations in the previously mentioned studies with conventional cigarettes might have differed.

In the current study, we observed that an extended duration of exposure to hookah smoke correlated with an augmented apoptotic response in the epithelial layer of the dorsal tongue. Comparable to these findings, other investigations have documented escalated apoptotic occurrences in samples of tongue squamous cell carcinoma and the absence of such occurrences in samples of normal tissues [27]. Furthermore, a progressive elevation in apoptotic indices was observed across normal oral mucosa, oral leukoplakia, and oral squamous cell carcinoma [10]. This finding suggests that hookah smoke exposure intensifies apoptotic processes, potentially implicating it in the initiation of carcinogenesis in the assessed mouse model.

In the current study, VEGFR-1 exhibited elevated expression in the epithelial layer of the dorsal tongue, with an increasing duration of exposure to hookah smoke. Similarly, another previous study revealed heightened VEGFR-1 expression in moderately differentiated oral squamous cell carcinoma (OSCC) lesions compared to well-differentiated and undifferentiated lesions, encompassing samples from the tongue, lip, and floor of the mouth [33]. Elevated VEGFR-1 expression has been documented in head and neck squamous cell carcinoma [34,35]. A prior study demonstrated that as dysplasia escalates, VEGFR-1 expression is progressively observed in deeper epithelial layers [11]. The heightened VEGFR-1 expression associated with hookah use and its increased expression in squamous cell carcinoma suggests that VEGFR-1 could potentially play a role in the early stages of carcinogenesis in the evaluated sample.

Aligned with the study’s objective to evaluate tissue protein effects during the initial periods of hookah use, therefore up to 30 days, changes in protein expression of VEGFR-1 and the apoptotic index were observed at 15 and/or 30 days. Further studies should be conducted to assess the long-term effects resulting from prolonged consumption.

Despite the limitations imposed by the ethics committee on the release of animals, the statistical power was high in the present study for results with statistical significance. A test with high statistical power could identify significant differences with greater precision, even when the sample size is limited, thereby ensuring the validity of the results obtained.

Some relevant considerations are that the methodology employed does not perfectly replicate the actual exposure to hookah smoke, as it was administered within a box rather than being directly placed in the mouse’s mouth. The smoke components settled on the mouse’s fur, and tongue exposure occurred as the mouse engaged in self-grooming. However, despite being an animal model, the histology of mouse tongues exhibits similarities to their human counterparts. Future studies investigating lesions in hookah users could be conducted to assess the expression of these markers [36].

## Conclusion

The implications of the findings for understanding the impact of hookah smoke on oral health should be clearly outlined: hookah smoke induced greater apoptosis and increased the expression of Vascular Endothelial Growth Factor Receptor 1 (VEGFR-1) in the epithelium of the tongue at 30 days, potentially playing a role in the initial stages of carcinogenesis in the early stages of hookah use.

## Supporting information

S1 Data **Data collected and used in the statistical analysis**.
(XLSX)
